# CircNr1h4 regulates the pathological process of renal injury in salt‐sensitive hypertensive mice by targeting miR‐155‐5p

**DOI:** 10.1111/jcmm.14863

**Published:** 2019-11-28

**Authors:** Chaosheng Lu, Bicheng Chen, Congcong Chen, Haiyan Li, Dan Wang, Yi Tan, Huachun Weng

**Affiliations:** ^1^ Department of Clinical Research & Department of Pediatrics The First Affiliated Hospital of Wenzhou Medical University Wenzhou China; ^2^ Department of Pharmacy Jinhua Central Hospital Jinhua China; ^3^ Department of Pharmaceutical Sciences Wenzhou Medical University Chashan University‐town Wenzhou China; ^4^ Departments of Pediatrics, Pharmacology and Toxicology Pediatric Research Institute University of Louisville Louisville KY USA

**Keywords:** circular RNAs, hypertension, miRNAs, renal injury

## Abstract

Circular RNAs are a class of widespread and diverse endogenous RNAs that may regulate gene expression in various diseases, but their regulation and function in hypertensive renal injury remain unclear. In this study, we generated ribosomal‐depleted RNA sequencing data from normal mouse kidneys and from injured mouse kidneys induced by deoxycorticosterone acetate‐salt hypertension and identified at least 4900 circRNA candidates. A total of 124 of these circRNAs were differentially expressed between the normal and injured kidneys. Furthermore, we characterized one abundant circRNA, termed circNr1h4, which is derived from the *Nr1h4* gene and significantly down‐regulated in the injured kidneys. RNA sequencing data and qPCR analysis also showed many microRNAs and mRNAs, including miR‐155‐5p and fatty acid reductase 1 (Far1), were differentially expressed between the normal and injured kidney and related to circNr1h4. In vitro, the silencing of circNr1h4 or overexpression of miR‐155‐5p significantly decreased Far1 levels and increased reactive oxygen species. Mechanistic investigations indicated that circNr1h4 acts as a competing endogenous RNA for miR‐155‐5p, leading to regulation of its target gene Far1. Our study provides novel insight into the molecular mechanisms underlying kidney injury in hypertension, which will be required to develop therapeutic strategies of targeting circRNAs for hypertensive kidney injury.

## 1 INTRODUCTION

Hypertension is a major public health problem with a high prevalence in populations, who have high dietary salt intake.^1^ Salt intake is one of the main factors that contribute to the development of hypertension.^2^, ^3^ Hypertension is a major risk factor for kidney failure, stroke and myocardial infarction.^4^ Furthermore, the kidneys play a key role in the pathogenesis of hypertension.^5^ A physiological defect in the kidneys impairs blood pressure‐induced sodium excretion, thus leading to salt‐sensitive hypertension.^6^ However, the mechanisms underlying the pathogenesis of salt‐sensitive hypertensive kidney disease remain unclear. Previous studies suggest that noncoding genomic regions are associated with genetically complex diseases, including hypertension.^7^ Noncoding RNAs, such as microRNAs (miRNAs), long noncoding RNAs and circular RNAs (circRNAs), have been reported to be associated with cardiovascular and renal diseases.^8^, ^9^

CircRNAs have recently been identified as a naturally occurring family of noncoding RNAs that are highly abundant in the eukaryotic transcriptome.^10^, ^11^ CircRNAs are characterized by covalently closed loop structures and have a stable structure.^12^, ^13^ CircRNAs were first described in 1991, and only a few circRNAs were identified over the following 20 years.^14^, ^15^, ^16^ However, high‐throughput sequencing and novel computational approaches have recently demonstrated their widespread and substantial presence within transcriptomes.^10^, ^11^, ^12^, ^13^ Furthermore, circRNA function is only currently beginning to be understood. ciRS‐7 has been shown to be a circRNA that sponges miR‐7, thus strongly suppresses miR‐7 activity.^12^ Additionally, a heart‐related circRNA sponges miR‐223 to protect the heart from cardiac hypertrophy and heart failure.^9^ A few studies have demonstrated that circRNAs are associated with hypertension and renal disease.^17^, ^18^ These studies all demonstrated that circRNAs play important roles in various diseases. However, their functions remain largely unknown and require further detailed investigations. Some studies demonstrate that circRNAs sponge miRNAs with miRNA response elements to sequester miRNAs and subsequently terminate the post‐transcriptional control of their target genes.^12^, ^19^

miRNAs are approximately 22‐25 nucleotides in length and act as negative regulators of their target mRNAs by promoting mRNA degradation or inhibiting mRNA translation.^20^, ^21^ Cumulating evidence has demonstrated that miRNAs play an important role in the regulation of renal development, physiology and pathology.^22^, ^23^, ^24^, ^25^, ^26^ MiR‐29b, miR‐107 and miR‐324‐3p are involved in the process of hypertensive nephropathy.^25^, ^26^ Additionally, miR‐214‐3p contributes to the development of salt‐sensitive hypertension in the kidney.^24^ Although these studies demonstrated that miRNAs are involved in the regulation of hypertensive nephropathy, upstream regulators of miRNAs that might be involved in hypertensive kidney injury remain largely unknown.

In this study, renal circRNA, miRNA and mRNA transcriptome analyses were performed in the kidneys of deoxycorticosterone acetate (DOCA)‐salt‐induced hypertensive mice and control mice. Furthermore, we characterized one abundant circRNA, termed circNr1h4, which is produced from the *Nr1h4* (nuclear receptor subfamily 1, group H, member 4) gene, that binds to miR‐155‐5p and acts as an endogenous miRNA sponge to regulate its target gene, which is fatty acid reductase 1 (Far1). Our study provides novel insight into the molecular mechanisms underlying kidney injury in salt‐sensitive hypertension.

## 2 MATERIALS AND METHODS

An expanded Materials and Methods is available at Supplemental Materials and Methods online.

### 2.1 Animal experiments and DOCA‐salt hypertension model

Adult male C57BL/6 mice (8‐10 weeks old) were purchased from the experimental Animal Center of Beijing University of Medical Science (Beijing, China) and allowed to acclimate for 2 weeks. Standard rodent chow and tap water were freely available throughout the experiments. All experiments were performed in accordance with the guidelines for Animal Experimentation of Wenzhou Medical University and approved by the Committee for Laboratory Animals. All surgeries were performed under isoflurane anaesthesia, and all efforts were made to minimize suffering.

We utilized a previously described DOCA‐salt mouse model^27^ with minor modifications. Unilateral nephrectomy was performed. After 1 week of recovery, a 21‐day‐releasing DOCA pellet containing 50 mg DOCA (Innovative Research of America) was implanted subcutaneously. Control animals underwent a sham operation. The animals (DOCA and control groups) were fed rodent chow and water containing 1% NaCl beginning on the third day before DOCA treatment. Urine was collected for 24 hours for the analysis of creatinine and albumin. Then, the mice were killed. Total RNA was extracted from the kidney with TRIzol reagent (Invitrogen) as previously described.^27^, ^28^

### 2.2 Cell culture and transfection

Mouse kidney collecting duct cells (M1 cells) were purchased from the Cell Resource of China and were tested and found to be negative for mycoplasma contamination before use. M1 cells were cultured in Dulbecco's modified Eagle's medium (DMEM) containing 4.5 g/L glucose/F‐12 and 10% foetal bovine serum. Transfection was performed with a ribo FECT CP Transfection Kit (RiboBio Co., Ltd.) according to the manufacturer's instructions. The miR‐155‐5p mimic, circNr1h4 inhibitor, mimic negative control and inhibitor negative control were purchased from RiboBio. M1 cells were transfected with 300 ng pCDNA3.1‐Far1 plasmid or negative control vector using Lipofectamine 3000 reagent (Invitrogen) in 24‐well plates for 48 hours. Transfection was performed according to the manufacturer's instructions. M1 cells were treated by 100 μmol/L palmitate for 18 hours and then were stained with dihydroethidium (DHE).

### 2.3 RNA sequencing analysis

For circRNAs and mRNA, the total RNA samples were treated with an Epicenter Ribo‐Zero Gold Kit (Illumina) to remove rRNA before constructing RNA‐seq libraries. The samples were fragmented and then synthesized as first‐ and second‐strand complementary DNA (cDNA) with random hexamer primers, dNTPs and DNA polymerase I using a PrimeScript RT reagent Kit (TaKaRa). The cDNA fragments were treated with T4 DNA polymerase to repair the ends and Klenow DNA polymerase to add ‐A and adapters at the 3′ end of the DNA fragments. The cDNA products were purified with AMPure XP beads and then subjected to PCR amplification. Then, the cleaved RNA fragments were reverse‐transcribed to create the final cDNA library in accordance with the mRNA‐Seq sample preparation kit protocol (Illumina). Then, we performed paired‐end sequencing on a HiSeq 4000 sequencing system (Illumina) following the manufacturer's recommended protocol.

For miRNAs, approximately 1 μg of total RNA was used to prepare a small RNA library according to the TruSeq Small RNA Sample Prep Kit protocol (Illumina). Then, we performed single‐end sequencing (1 × 50 bp) on an Illumina Hiseq 2500 sequencing system following the manufacturer's recommended protocol.

### 2.4 Analysis of raw sequencing data

For circRNAs, FastQC (http://www.bioinformatics.babraham.ac.uk/projects/fastqc/) was used to verify sequence quality. Bowtie2^29^ and TopHat2^30^ were used to map the reads to the mouse genome. The remaining reads (unmapped reads) were mapped to the genome using TopHat‐Fusion.^31^ First, CIRCexplorer was used to assemble the mapped reads to circular RNAs de novo*.* 32 Then, back‐spliced reads were identified in unmapped reads by TopHat‐Fusion and CIRCexplorer. All samples generated unique circular RNAs. We normalized circRNA contents as the number of uniquely mapped fragments per kilobase of exon per million fragments mapped (FPKM). According to the grouping of samples, the default threshold values of significantly different genes were typically set as follows: |log2*fold change*| ≥ 1 and *P*‐value ≤ .05. According to the similarity of gene expression profiles of the samples, cluster analysis was carried out to intuitively show the expression of genes in the different samples (or different treatments) to obtain relevant biological information. The differentially expressed gene FPKM was expressed by a *z*‐value that was calculated as follows: *Z* sample‐i = [(log2 (signal sample ‐ i) − Mean (log2 (signal) of all samples)]/[standard deviation (log2 (signal) of all samples)].

For miRNAs, raw reads were subjected to the in‐house software ACGT101‐miR (LC Sciences) to remove junk, low complexity, adapter dimers, common RNA families (snRNA, snoRNA, tRNA, rRNA) and repeats. Subsequently, unique sequences with 18‐26 nucleotide in length were mapped to mouse precursors in miRBase 22.0 by a BLAST search to identify known miRNAs and novel 3p‐ and 5p‐ derived miRNAs. Unique sequences mapped to mature mouse miRNAs in hairpin arms were identified as known miRNAs. Unique sequences mapped to the other arm of known specific species precursor hairpin opposite to the annotated mature miRNA‐containing arm were considered to be novel 5p‐ or 3p‐derived miRNA candidates. The remaining sequences were mapped to other selected species precursors (with the exclusion of specific species) in miRBase 22.0 by a BLAST search, and the mapped pre‐miRNAs were further BLASTed against specific species genomes to determine their genomic locations.

We constructed an mRNA‐miRNA‐circRNA interaction network based on miRNA seed sequence binding sites that we detected in the mRNA and circRNA sequences. The interactions of miRNA‐mRNA and miRNA‐circRNA were predicted by TargetScan 50 (http://www.targetscan.org/), and Miranda 3.3a (http://www.microrna.org). Finally, the data predicted by both algorithms were combined, and the overlaps were calculated.

Gene ontology (GO) analysis (http://www.geneontology.org) was used to characterize circRNA‐hosting genes. GO terms provide information, such as the molecular function, cellular component, and biological process, about the biological processes in which genes are involved. Kyoto Encyclopaedia of Genes and Genomes (KEGG; http://www.kegg.jp) pathway analysis were also performed to provide insights into the molecular interaction and reaction networks of circRNAs and miRNAs that were differentially expressed using Visualization and Integrated Discovery (DAVID, version 6.7; http://david.ncifcrf.gov).

### 2.5 Luciferase reporter assays

The wild‐type and mutated 3′UTR of the Far1 gene or circNr1h4 was amplified by PCR and inserted into Firefly luciferase reporter pmirGLO vector (Promega). HEK293T cells were cotransfected with 300 ng Far1‐pmirGLO or circNr1h4‐pmirGLO and 50 nmol/L miR‐155‐5p mimic or mimic negative control using Lipofectamine 3000 reagent (Invitrogen) in 24‐well plates for 48 hours. The activation of Firefly and Renilla luciferase was analysed by a dual‐luciferase reporter assay kit (Promega) according to the manufacturer's instructions.

### 2.6 Statistical analysis

The results were presented as the mean ± SE. The data were analysed using one‐way analysis of variance (ANOVA) and an unpaired two‐tailed Student's *t* test. All analyses were performed using SPSS 22.0. Differences were considered significant at *P* < .05.

## 3 RESULTS

### 3.1 Hypertensive renal damage induced by DOCA‐salt

**Figure 1:**
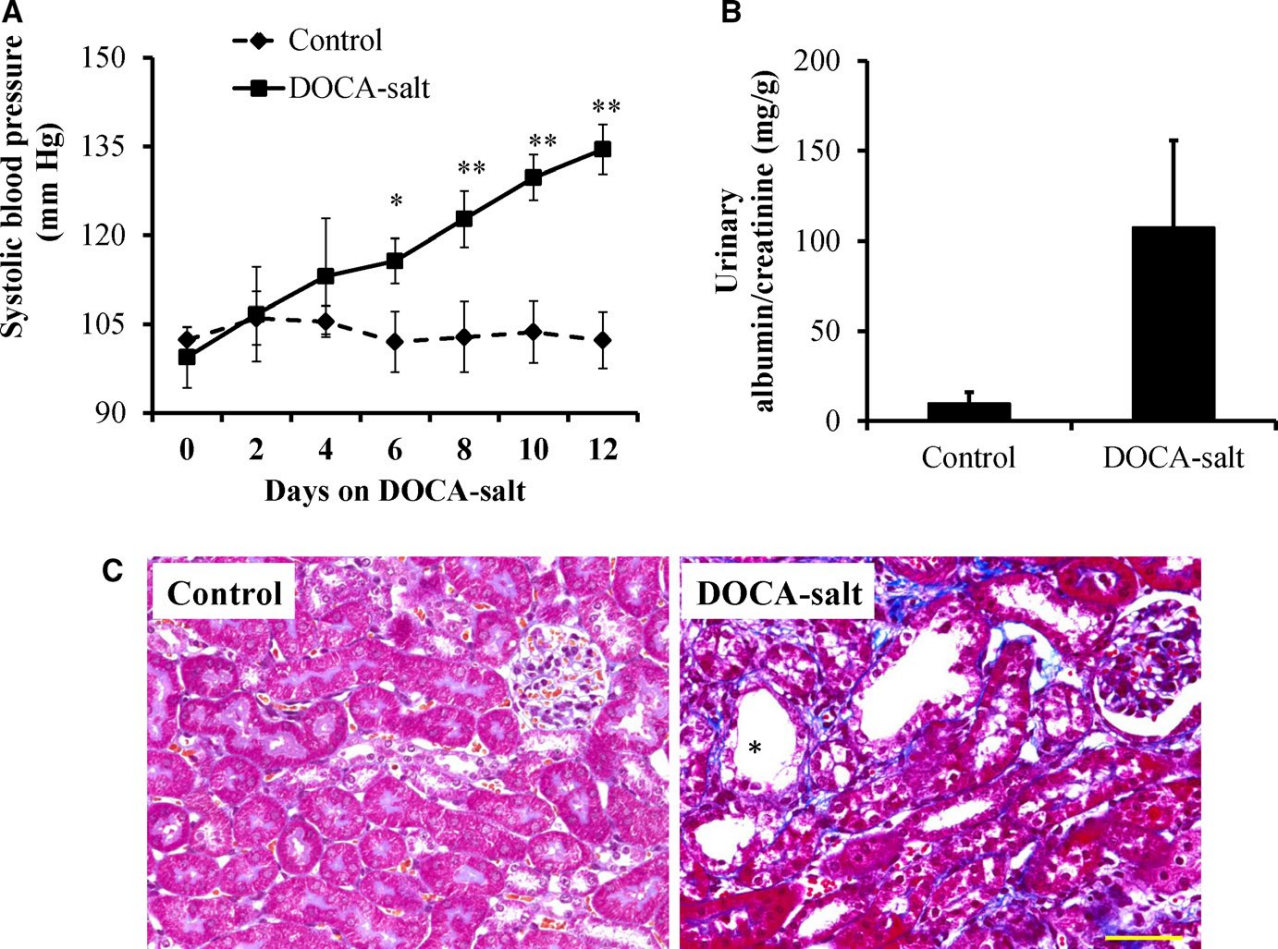
Deoxycorticosterone acetate (DOCA)‐salt treatment induced hypertension, albuminuria and kidney injury. (A) The systolic blood pressure (SBP) in DOCA‐salt‐induced hypertensive mice was monitored by a tail‐cuff. Urinary albumin (B) levels were elevated by treatment with DOCA for 14 d. The values are presented as the mean ± SE, n = 6 mice/group, *P < .05, **P < .01 vs the control group. (C) Representative images of Masson's trichrome‐stained kidney sections from the control and DOCA‐salt‐treated mice showed gradually aggravated tubular dilatation/atrophy (*), epithelial cell necrosis, interstitial oedema and fibrosis (blue). Scale bar: 50 μm

The DOCA‐salt treatment significantly increased blood pressure and resulted in significantly higher level of urinary albumin compared to the controls (Figure 1A,B). The morphology in the kidneys of mice treated with DOCA‐salt for 14 days was assessed. An increase in renal tubular lesions was observed in DOCA‐salt‐treated mice compared with the control mice. Masson's trichrome staining showed that the cortical area of mice treated with DOCA‐salt showed more severe tubulointerstitial fibrosis compared with the controls (Figure 1C). These results verified that DOCA‐salt treatment for 14 days achieves hypertensive renal damage.

### 3.2 Profiling of circRNAs in the kidneys of control and DOCA‐salt‐induced hypertensive mice

**Figure 2:**
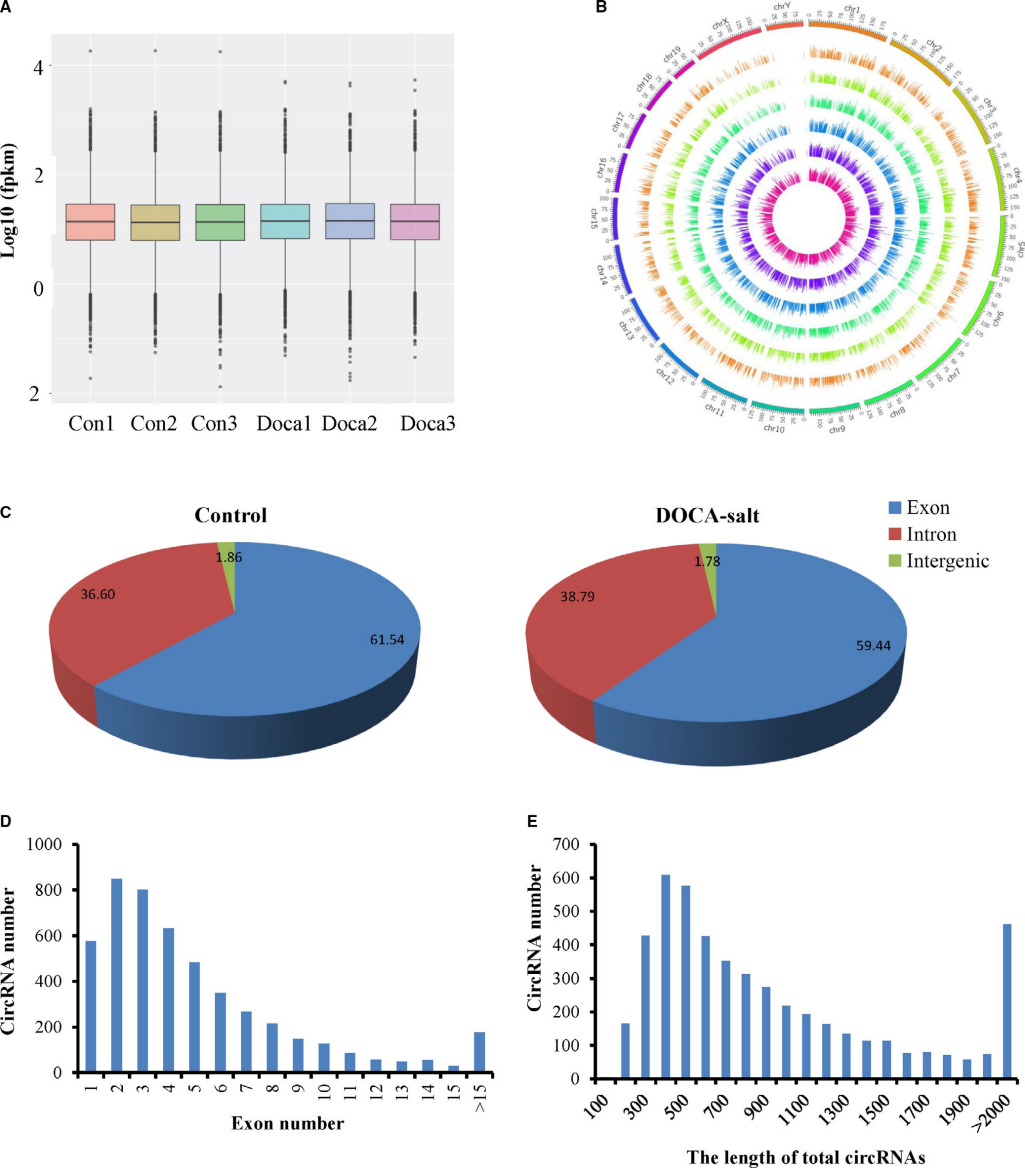
Characteristics of circRNA expression in the kidneys of the control and DOCA‐salt‐induced hypertensive mice. (A) Box plots showing the distribution of expression levels, and (B) circus plot showing the distribution of circRNAs in different chromosomes for all samples. (C) The origin of circRNAs described in the mouse kidney and (D) circRNAs containing varying numbers of exons. (E) The size distribution of circRNAs in the kidneys of the control mice and the mice treated with DOCA‐salt for 14 d

CircRNA transcripts were characterized by using RNA sequencing (RNA‐seq) analysis of ribosomal RNA‐depleted total RNA from the renal tissues of 3 control and 3 DOCA‐salt‐induced hypertensive mice. ~90 million reads from each sample were mapped to the mouse reference genome by TopHat2. A detailed summary for all the samples is shown in Table S1. We identified 4904 circRNA candidates, which included 4589 circular exonic RNAs, containing at least one unique back‐spliced read (Table S2). Box plots showed a similar distribution of normalized expression levels for each sample (Figure 2A). The genomic loci from which circRNAs are derived were widely distributed across chromosomes except the Y chromosome (Figure 2B). The distribution of circRNAs between different chromosomes was not uniform; furthermore, there was a trend in which the number of circRNAs per chromosome increased with the absolute chromosome length. Most mapped sequence reads were derived from exonic regions (≈60%), but there were no noticeable differences between the DOCA‐salt mice and control mice (Figure 2C). Most circRNAs contained one to ten exons, while approximately 9.2% of circRNAs contained more than ten exons (Figure 2D). The length of most exonic circRNAs ranged from 200 to 2000 nucleotide (nt), and the median length was approximately 700 nt (Figure 2E).

### 3.3 Identification of differentially expressed circRNA

**Figure 3:**
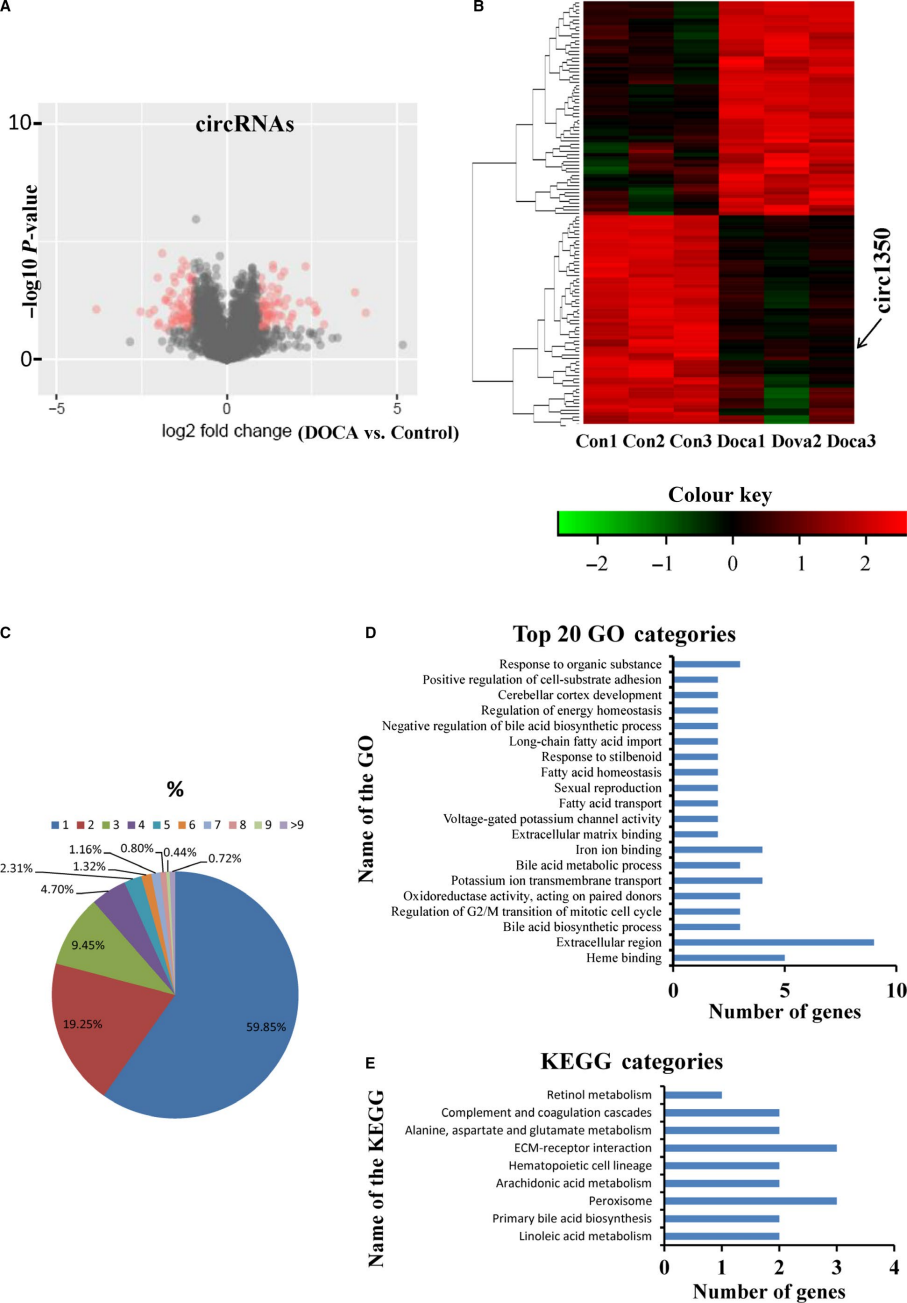
Differential expression of circRNAs and its correlation and regulation with their parental linear transcripts in the kidneys of the control and DOCA‐salt‐induced hypertensive mice. (A) Volcano plots showing the significant up‐regulated and down‐regulated circRNAs, (B) A cluster analysis heat map showing 124 differentially expressed circRNAs in the kidneys of the control mice and the mice treated with DOCA‐salt for 14 d. n = 3 mice/group. (C) The numbers of circRNAs produced by the same gene. Gene ontology (D) and Kyoto Encyclopaedia of Genes (KEGG) terms (E) of host genes from which differentially expressed circRNAs were identified

Based on the expression of circRNA analysis (FPKM), 124 circRNAs were significantly (*P* < .05) differently expressed in the kidney of the DOCA‐salt mice compared with the control mice (Table S2). There were 62 up‐regulated and 62 down‐regulated circRNAs in the kidney of the DOCA‐salt mice compared with the control mice, as shown in the volcano plots (Figure 3A). CircRNA267 (FPKM = 4338.4) expression level of all up‐regulated circRNAs was the highest (Table S3), and circRNA2748 (FPKM = 18 255.1) had the highest expression level of all down‐regulated circRNAs (Table S4). Specifically, by using a *P*‐value cut‐off .05 and a fold‐change cut off 2.0, a cluster analysis revealed differential circRNA expression in the kidneys between the DOCA‐salt mice and control mice (Figure 3B).

### 3.4 Correlation and regulation between circRNAs and their parental transcripts

To understand the function of circRNAs, we analysed circRNA number from their host genes and found that one parental gene generated many circRNA isoforms. The 4904 circRNAs identified in the kidneys of the DOCA‐salt mice and control mice originated from only 2509 host genes (Table S5). A notable example is the Pkhd1, which generated 72 distinct circRNAs (at least one unique back‐spliced read). Approximately 80% of the host genes produced one or two circRNA isoforms (Figure 3C). To further understand the potential functions of circRNAs in the process of salt‐sensitive hypertensive kidney injury, we compared the expression patterns of circRNAs to the patterns, such as up‐regulation, down‐regulation and similar expression patterns, of their host genes. Our results showed that circRNAs with a differential pattern of altered expression levels were comparable with their parental genes in the regulation of hypertensive kidney injury. We found only 11 of 62 circRNAs that were up‐regulated when comparing the DOCA‐salt mice with the control mice that were accompanied by similar changes in the expression of the corresponding parental mRNA (10 genes, Table S6). Furthermore, only 8 of 62 circRNAs that were down‐regulated were accompanied by similarly down‐regulated corresponding paternal mRNAs (7 genes). Approximately 105 significant differential circRNAs had no similar patterns of mRNA expression in the aetiological process of salt‐sensitive hypertensive kidney injury, indicating that circRNAs may have differential function compared to their host genes.

To further investigate the function of circRNAs, GO enrichment analysis was performed. We found significant enrichment of 267 functional groups (*P* < .05). The 20 most significant functional annotations, including heme binding‐ and fatty acid metabolism‐related groups, are shown in Figure 3D. KEGG pathway enrichment analysis was performed to further understand the molecular interactions and biological functions of genes hosting significantly differentially expressed circRNAs. The 9 significant KEGG pathways may be involved in the aetiological process of salt‐sensitive hypertensive kidney injury, are shown in Figure 3E. Linoleic acid metabolism including 24 annotated genes was found to have the highest level of significance.

### 3.5 Validation of circRNA expression by qPCR

**Figure 4:**
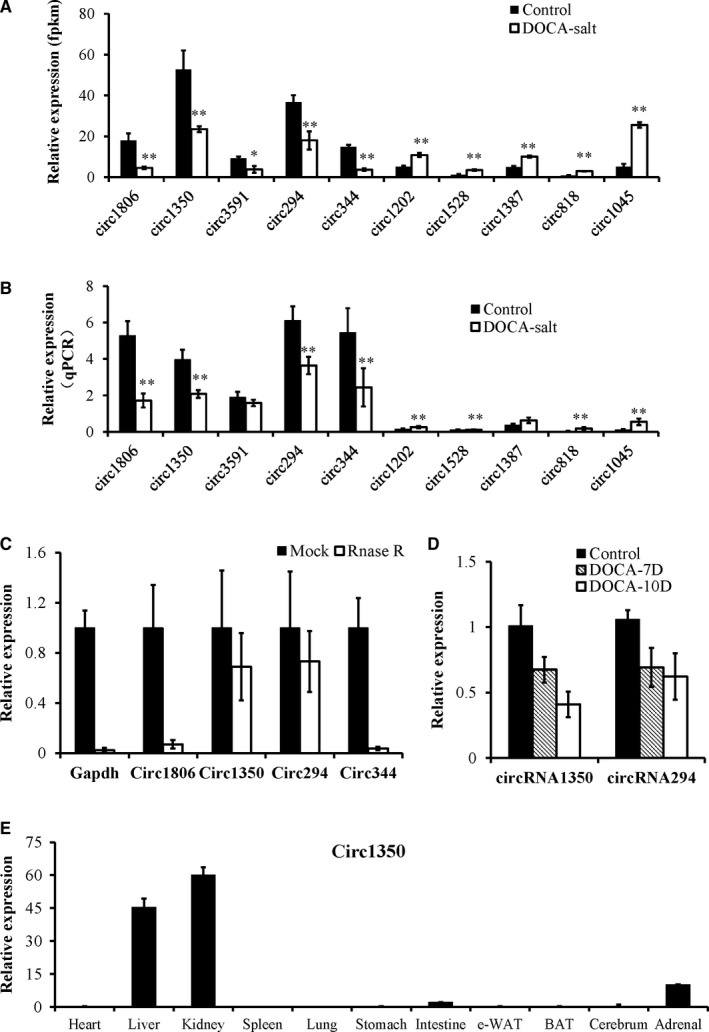
Validation of differentially expressed circRNAs by qPCR. (A) Ten circRNAs that were selected from our RNA sequencing approach and that exhibited significantly different expression patterns. (B) Validation of the differential expression of circRNAs in the kidneys between the control mice and the mice treated with DOCA‐salt for 14 d using qPCR. (C) qPCR analysis of the expression levels of circ1350, circ1806, circ294, circ344 and linear RNA (Gapdh) treated with RNase R. (D) The expression levels of circRNA1350 and circRNA294 were dependent on the time of the DOCA‐salt treatment. (E) The expression levels of circRNA1350 in different tissues of the mice. The values are presented as the mean ± SE, n = 6 mice/group, *P < .05, **P < .01 vs the control group

The physical properties of these circRNAs were examined to verify that the back‐spiced events were indicative of truly circular RNA (and not linear RNA). Outward‐facing primers were designed against 10 circRNAs (at least contain two unique back‐spliced reads) selected from 124 differentially expressed circRNA transcript candidates (Figure 4A). The qPCR results showed the presumed patterns of up‐regulation and down‐regulation of the 10 circRNAs that were distinctly similar in both types of analyses (Figure 4B). The enrichment of 4 back‐spliced events (candidates with higher expression levels) was validated following Rnase R treatment, whereas the expression levels of linear RNAs (Gapdh) and 2 candidates were remarkably decreased. However, the expression levels of circRNA1350 and circRNA294 were not obviously decreased, indicating they conformed to the characteristics of circular RNA (Figure 4C). Furthermore, the expressed levels of circRNA1350 and circRNA294 were dependent on the time of the DOCA‐salt treatment, and circRNA1350 was more obvious (Figure 4D). We also assessed the expressed levels of circRNA1350 in different tissues and found that circRNA1350 was more highly expressed in the kidneys and liver (Figure 4E).

### 3.6 Differential miRNA and mRNA expression in the kidneys between the control mice and DOCA‐salt‐induced hypertensive mice

**Figure 5:**
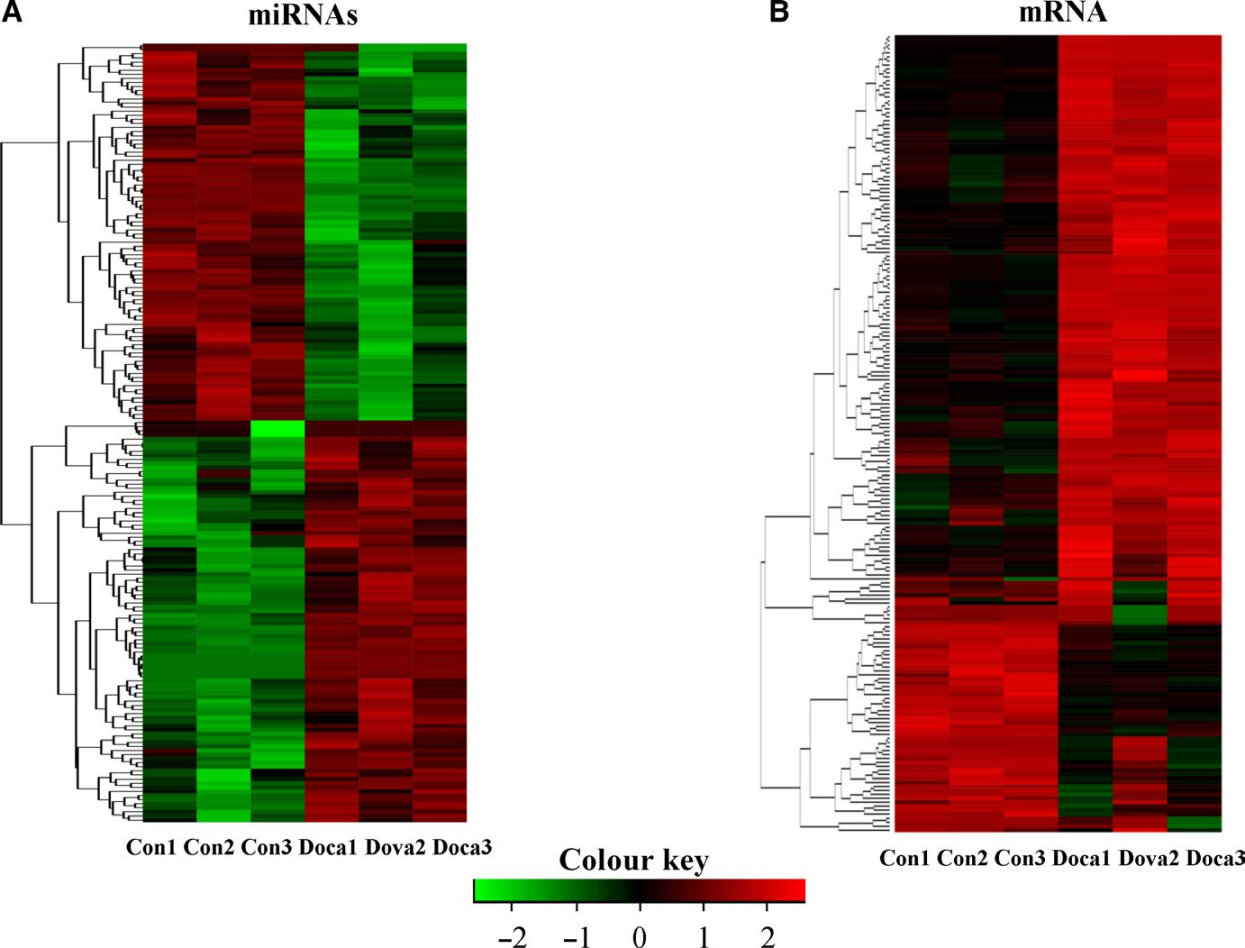
Differential expression of miRNAs and mRNA in the kidneys of the control and DOCA‐salt‐induced hypertensive mice. Cluster analysis heat maps showing 190 differentially expressed miRNAs (A) and 504 differentially expressed mRNAs (B) in the kidneys of the control mice and the mice treated with DOCA‐salt for 14 d. n = 3 mice/group

Previous studies have indicated that circRNAs contribute to the development of various diseases through miRNAs and their target genes.^9^, ^12^ We also characterized miRNAs and mRNA using RNA sequencing analysis from the kidneys of the DOCA‐salt treated and control mice. We found 1094 miRNAs expressed in the kidneys of the DOCA‐salt treated and control mice and 726 miRNAs mapped to previously published databases obtained from miRbase (mouse species) (Table S7). There were 98 miRNAs that were significantly up‐regulated and 92 miRNAs that were significantly down‐regulated. Cluster analysis revealed distinctly different miRNA expression in the kidneys between the DOCA‐salt mice and control mice, as shown in Figure 5A. We also found 21 404 genes expressed in the kidneys of the DOCA‐salt treated and control mice, and there were 504 significantly differentially expressed genes (Table S8). A total of 504 significantly differentially expressed genes (143 down‐regulated genes and 361 up‐regulated genes) were analysed. The cluster analysis heat map revealed distinctly different gene expression patterns in the kidneys between the DOCA‐salt mice and control mice, as shown in Figure 5B.

### 3.7 circNr1h4 acts as a competing endogenous RNA for miR‐155‐5p and then regulates its target gene Far1

**Figure 6:**
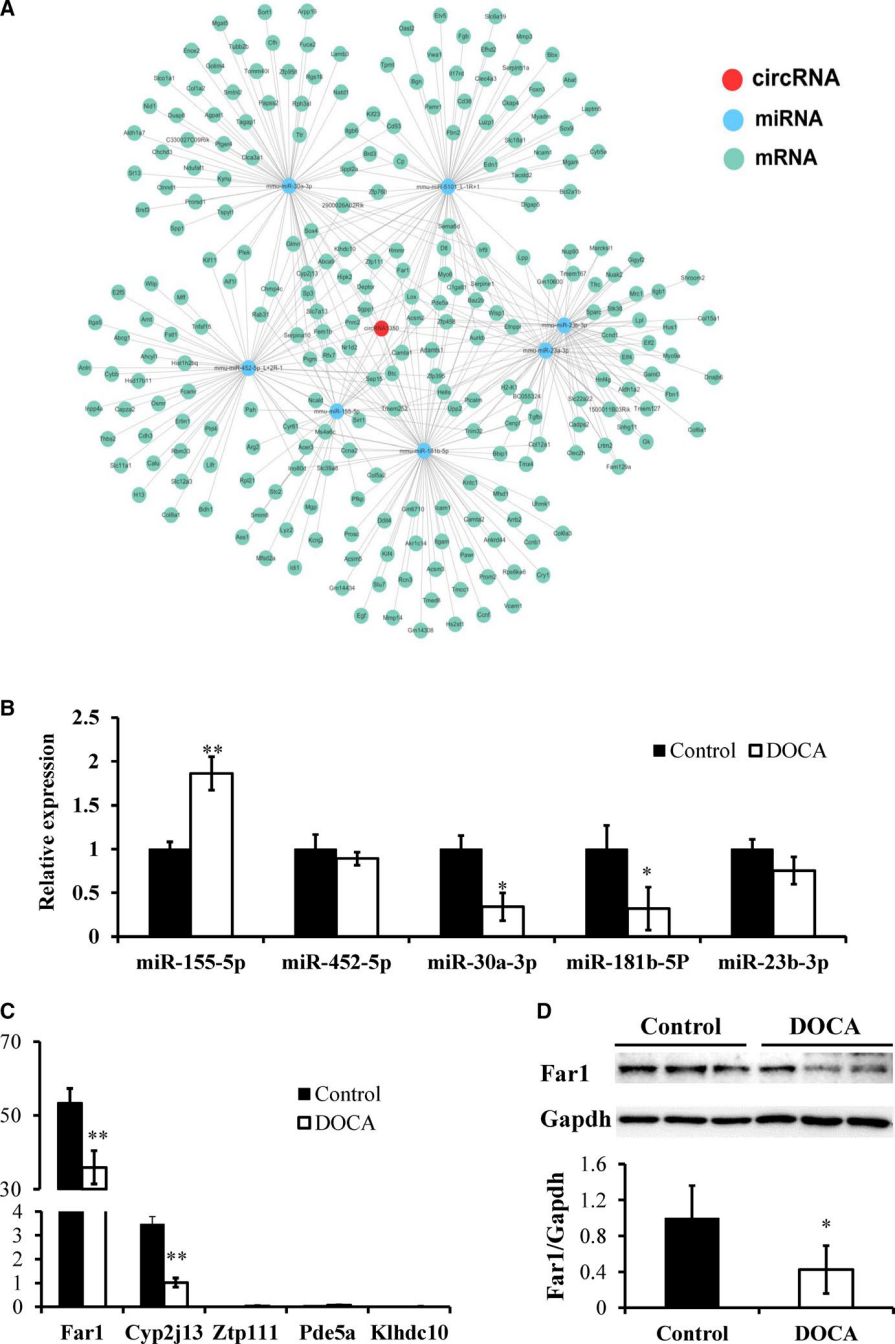
circRNA‐miRNA‐mRNA network in the kidneys of the control and DOCA‐salt‐induced hypertensive mice. (A) The predicted circNr1h4‐miRNA‐mRNA network. Validation of the differential expression of miRNAs (B) and mRNAs (C) in the kidneys between the control mice and the mice treated with DOCA‐salt for 14 d using qPCR. (D) Western blot analysis of Far1 expression in the kidneys. The values are presented as the mean ± SE, n = 6 mice/group, *P < .05 and **P < .01 vs the control group

Next, we focused on one circRNA (circRNA1350), which showed the highest expression levels in the kidney and significant differential expression between the control mice and DOCA‐salt‐treated mice, suggesting a potential role in the process of hypertensive kidney injury. We named circNr1h4 based on its host gene *Nr1h4* (nuclear receptor subfamily 1, group H, member 4), which is located on chromosome 10. Based on shared binding sites in circRNAs, miRNAs and mRNAs (TargetScan score > 50 and Miranda energy < −10), we constructed a circRNA‐miRNA‐mRNA network, as shown in Figure 6A. We found that 7 miRNAs were differentially expressed between the control mice and DOCA‐salt‐treated mice that were predicted to bind to circNr1h4. The qPCR results validated that miR‐155‐5p was significantly up‐regulated and miR‐30a‐3p and miR‐181b‐5p were significantly down‐regulated after inducing hypertensive kidney injury (Figure 6B), similar to the RNA sequencing data. As shown in Figure 6A, 30 genes may be the target genes of miR‐155‐5p. Previous studies have indicated that miRNAs target the 3′UTR of genes to inhibit their expression; therefore, we focused on the 13 down‐regulated genes, including Far1. qPCR analysis validated that Far1 was the highest expressed in the kidneys and was significantly down‐regulated after inducing hypertensive kidney injury (Figure 6C), similar to the RNA sequencing data. The Western blotting results also showed that the Far1 levels of the DOCA‐salt mice were significantly less than those of the control mice (Figure 6D).

**Figure 7:**
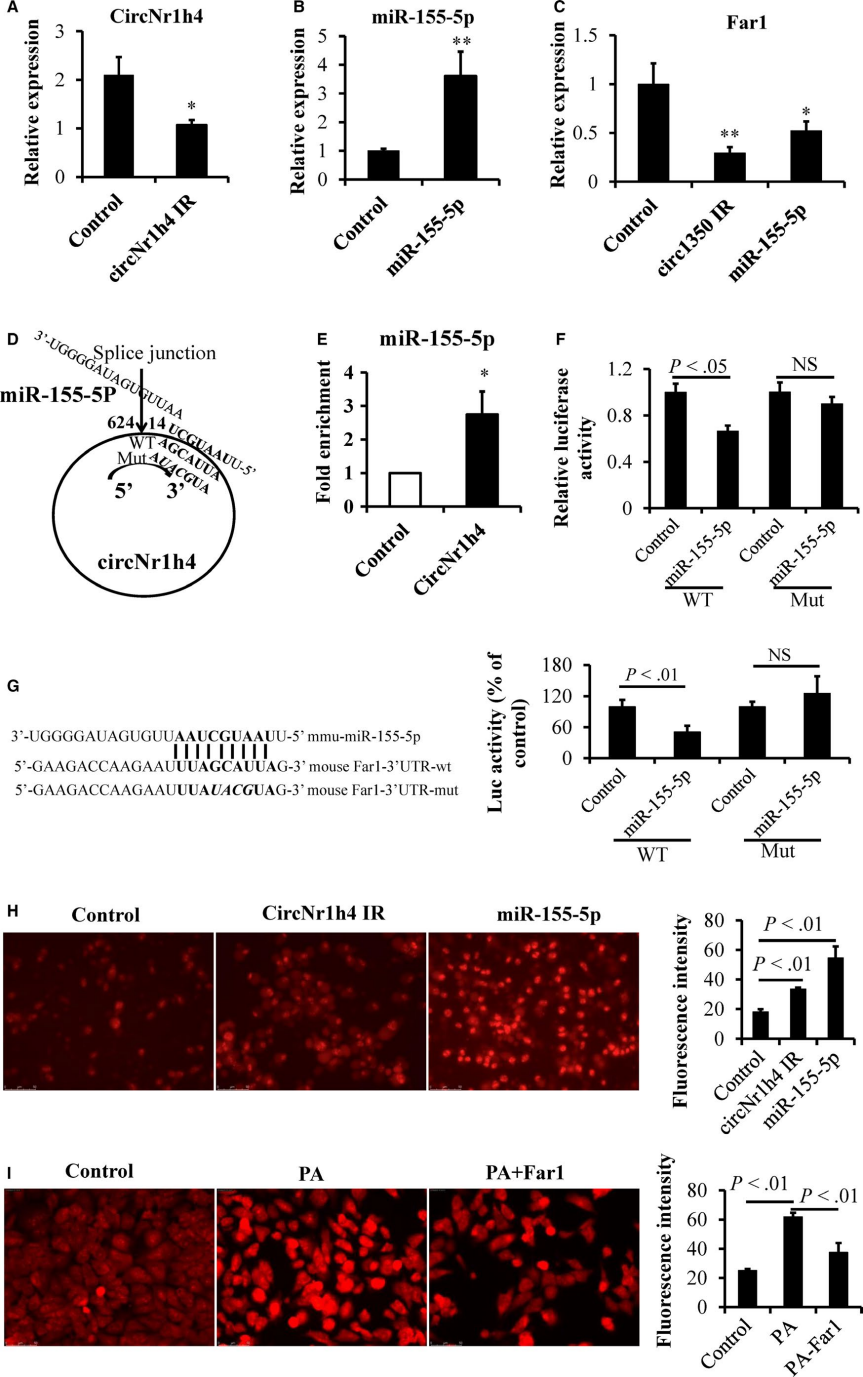
circNr1h4 regulates Far1 by sponging miR‐155‐5p. (A) The circNr1h4 inhibitor (IR) significantly decreased the expression level of circNr1h4, and (B) the miR‐155‐5p mimic significantly increased the expression level of miR‐155‐5p in mouse kidney collecting duct cells (M1). (C) The circNr1h4 IR or miR‐155‐5p mimic significantly decreased Far1 expression levels in M1 cells. (D) CircNr1h4 contains one site that is complementary to miR‐155‐5p, as predicted by TargetScan. (E) miR‐155‐5p was pulled down by the circNr1h4 probe, and miR‐155‐5p was analysed by qPCR. (F) miR‐155‐5p inhibits luciferase activities from the wild‐type pmirGLO‐circNr1h4 vector. (G) The miR‐155‐5p binding site in the Far1 3′UTR is shown; miR‐155‐5p inhibits luciferase activities from the wild‐type pmirGLO‐Far1 vector. (H) Representative images of M1 cells were stained with dihydroethidium (DHE) after the circNr1h4 inhibitor or miR‐155‐5p mimic treatment for 24 h, Fluorescence intensity of 50 cells from each sample was measured. (I) Representative images of M1 cells were stained with DHE after palmitate (PA) treatment for 18 h or/and Far1 overexpression treatment for 48 h. Fluorescence intensity of 50 cells from each sample was measured. Scale bar: 50 μm; n = 3 replicates/group; *P < .05 and **P < .01 vs the control group; NS indicates no significant difference

Fluorescence in situ hybridization (FISH) showed that circNr1h4 was most highly expressed in the tubular cells of kidneys (Figure S1), so we transfected mouse M1 cells with a circNr1h4 inhibitor to decrease circNr1h4 levels (Figure 7A). We also transfected M1 cells with a miR‐155‐5p mimic to increase miR‐155‐5p expression levels (Figure 7B). As expected, miR‐155‐5p overexpression or circNr1h4 knockdown significantly decreased the expression level of Far1 (Figure 7C), which may be because of miR‐155‐5p being released from circNr1h4 and inhibiting Far1. Based on the competing endogenous RNA analysis, we speculated that circNr1h4 may competitively bind to miR‐155‐5p and regulate its expression. Using TargetScan prediction program, miR‐155‐5p was found to contain a putative binding site of circNr1h4, as shown in Figure 7D. To verify this result, we used biotin‐coupled circNr1h4 to investigate whether circNr1h4 can pull down miR‐155‐5p. We observed a greater enrichment of miR‐155‐5p in the circNr1h4‐captured fraction compared with the negative control (Figure 7E). Furthermore, luciferase reporter assay validated that miR‐155‐5p inhibits luciferase activities from the wild‐type pmirGLO‐circNr1h4 vectors but not from the mutated pmirGLO‐circNr1h4 (Figure 7F). These results suggest that circNr1h4 may function as a sponge to miR155‐5p.

Furthermore, using TargetScan prediction program, we found that the 3′UTR of Far1 contains a potential binding site for miR‐155‐5p. Luciferase reporter assay also showed that miR‐155‐5p inhibits luciferase activities from the wild‐type pmirGLO‐Far1 vector but not from the mutated pmirGLO‐Far1 (Figure 7G). These results suggest that circNr1h4 acts as a sponge to miR‐155‐5p and mitigate the inhibitory effect of miR‐155‐5p on the expression of Far1. DHE staining showed that the silencing of circNr1h4 or miR‐155‐5p overexpression in M1 cells significantly increased the levels of reactive oxygen species (ROS), which may be because of decreased Far1 expression (Figure 7H). Furthermore, exposure of M1 cells to palmitate induced a significant decrease of Far1 (Figure S2) and a significant increase in ROS (DHE staining, Figure 7I). Furthermore, Far1 overexpressing plasmid restored the decreased levels of Far1 and significantly inhibited the increased levels of ROS induced by palmitate treatment (Figure 7I). Our results suggested that Far1 may play a role in the redox equilibrium in the hypertensive kidney injury.

## 4 DISCUSSION

Our present study identified a large number of circRNAs in normal and DOCA‐salt‐induced hypertensive mouse kidneys from diverse genomic locations, and numerous abundant circRNAs were found to be differentially expressed in the injured kidneys compared with the normal kidneys, suggesting that they may play an important role in the process of hypertensive kidney injury. Our data demonstrated that circNr1h4 may act as a regulator of Far1 by sponging miR‐155‐5p in hypertensive kidney injury. Our results provide new insight into the pathogenesis of DOCA‐salt‐induced hypertensive kidney injury.

Our results show that circRNAs are mostly derived from exon region of precursor mRNA, indicating that precursor mRNAs can produce a variety of additional transcripts. CircRNAs do not contain a polyA tail and are RNase R‐resistant, providing an opportunity to distinguish between mRNAs. Accumulating evidence has suggested that circRNAs are not simple products of splicing errors or mis‐splicing, and some studies have reported that many circRNAs may play an important role in neural development and epithelial‐mesenchymal transition.^33^, ^34^ Our results showed that the expression pattern of circRNAs is different from their host gene, suggesting that they possess differential effects on regulating DOCA‐salt‐induced hypertensive kidney injury that is distinct from linear RNAs. Our data showed that there are 124 circRNA transcript candidates that are differentially expressed (*P* < .05, threshold > 2‐fold) in injured kidney compared with normal kidney, and 10 selected circRNAs showed at least two unique back‐spliced reads. RNase R treatment and qPCR analysis using outward‐facing primers further validates circNr1h4 is truly circular RNA. Furthermore, circNr1h4 is the highest expression levels in the kidney and significant differential expression between the control mice and DOCA‐salt‐treated mice, suggesting a potential role in the process of hypertensive kidney injury.

Many studies have shown that circRNAs may act as endogenous sponge RNAs to bind miRNAs and regulate the expression of miRNA target genes.^9^, ^12^, ^13^, ^35^, ^36^ One report showed that circHIPK3 regulates cell growth by sponging multiple miRNAs, specifically miR‐124.^35^ Wang and his colleagues reported that the circRNA HRCR sponges miR‐223 to inhibit its activity and then protect the heart from cardiac hypertrophy and heart failure.^9^ A study reported by Hansen et al^12^ showed that the ciRs‐7 functions as a miR‐7 sponge to inhibit its activity, increasing expression levels of miR‐7 target genes. Another study showed that the circRNA CDR1as acts as a miR‐7 sponge in neuronal tissues and sequesters miR‐7 from its target genes.^13^ Recently, Wei and his colleagues' study showed that circLMO7 promotes myoblast proliferation and protects them from apoptosis by sponging miR‐378a‐3p.^36^ These studies demonstrated that miRNA sponge effects achieved by circRNAs are a typical phenomenon in many diseases. Consistent with previous studies, our study showed that circNr1h4 regulates the expression levels of Far1 by sponging miR‐155‐5p and is involves in DOCA‐salt‐induced hypertensive kidney injury.

A single miRNA can target many mRNAs by binding its 3′UTR.^37^, ^38^ Previous studies have shown that the proapoptotic programmed cell death protein 4 is inhibited by miR‐21 in acute kidney injury (AKI).^39^, ^40^ miR‐21 also suppresses LC3‐II and beclin‐1 induction in ischaemia‐reperfusion‐injured kidney by targeting Rab11a.^41^ Liu and his colleagues found that renal miR‐214‐3p is involved in the development of hypertension that might be partially mediated by targeting eNOS.^42^ Another study showed that miR‐214 targets phosphatase and tensin homolog to promote the AKT signalling pathway and then increases cell proliferation and survival.^43^ These studies indicated that one miRNA can have multiple targets and functions in the kidney. Some studies have shown that miR‐155 plays an important role in several type of kidney injury. For example, the miR‐155 expression level is increased in ischaemic and cisplatin‐induced AKI, and it inhibits c‐Fos to regulate cell apoptosis.^44^ MiR‐155 directly targets FoxO3a and regulates renal tubular cell apoptosis, which is involved in renal ischaemia‐reperfusion injury.^45^ Another report also showed that miR‐155 is a positive regulator of the nucleotide‐binding domain like receptor protein 3 (NLRP3) pathway by inhibiting the targeted FOXO3a gene, and inhibition of the NLRP3 inflammasome ameliorates renal damage.^46^ MiR‐155 can also bind to the 3′UTR of Sirt1 to reduce its expression and decrease the expression of LC3‐II and ATG5 in renal tubular injury of diabetic kidney disease.^47^ For the first time, our current study demonstrated that miR‐155‐5p targets Far1, which is a protein that is highly expressed in the kidney and converts a fatty acyl‐CoA into a fatty alcohol and CoA‐SH, and circNr1h4 likely regulates Far1 perhaps by sponging miR‐155‐5p.

Far1 is a rate‐limiting enzyme in plasmalogen synthesis and is enriched in subcellular fractions containing catalase,^48^, ^49^ which is a peroxisome marker enzyme. Far1 deficiency results in reduced plasmalogens and a peroxisomal disorder associated severe intellectual disability, epilepsy and cataracts.^50^ Plasmalogens are essential membrane components that have many diverse roles, including the protection against ROS injury.^51^ Sindelar et al^52^ showed that the oxidative products of plasmalogens generated by various free radicals and singlet oxygen are unable to further propagate lipid peroxidation, suggesting plasmalogens may terminate lipid oxidation. The provision of chimyl alcohol to hearts has been shown to reduce reperfusion injury following ischaemia in rats, as indicated by reduced creatine kinase release and decreased lipid peroxidation and then increased left ventricular function and coronary flow, suggesting that increased plasmalogen levels might protect again ischaemic damage.^53^ These studies suggested that Far1 plays a role in many physiological and pathological processes by mediating plasmalogen and the redox equilibrium. Our results further showed that circNr1h4 regulates Far1 and ROS by targeting miR‐155‐5p, which may be involved in the pathological process of hypertensive kidney injury.

In conclusion, the circNr1h4‐miR‐155‐5p‐Far1 axis is an important regulatory network involved in DOCA‐salt‐induced hypertensive kidney injury. The detailed regulatory mechanism in the kidney requires further investigation.

## CONFLICT OF INTEREST

The authors confirm that there is no conflict of interests.

## **AUTHORS**'** CONTRIBUTIONS**

LCS, CBC and WHC performed the research; CCC, LHY and WD analysed the data; TY revised the manuscript; and WHC designed the research study and wrote the paper.

## Data Availability

The data used to support the findings of this study are available from the corresponding author upon request.
