# Corrigendum

**DOI:** 10.1111/jcmm.15094

**Published:** 2020-04-22

In the article,^1^ the authors have reported a correction to the affiliation of Yi Tan on page 3560. The affiliation, Department of Pharmaceutical Sciences, Wenzhou Medical University, Chashan University Town, Wenzhou, China, should have been removed from the author byline.

The author byline reads:

Yi Tan^4^

^3^Department of Pharmaceutical Sciences, Wenzhou Medical University, Chashan University‐town, Wenzhou, China.

^4^Departments of Pediatrics, Pharmacology and Toxicology, Pediatric Research Institute, University of Louisville, Louisville, KY, USA.

The author byline should have read:

Yi Tan^3^

^3^Departments of Pediatrics, Pharmacology and Toxicology, Pediatric Research Institute, University of Louisville, Louisville, KY, USA.

## References

[1] Lu C , Chen B , Chen C , et al. CircNr1h4 regulates the pathological process of renal injury in salt‐sensitive hypertensive mice by targeting miR‐155‐5p. J Cell Mol Med. 2020;24:1700‐1712. 31782248 10.1111/jcmm.14863 PMC6991678

