# Supplementary figures and images for: CircNr1h4 regulates the pathological process of renal injury in salt‐sensitive hypertensive mice by targeting miR‐155‐5p
Source: J Cell Mol Med. 2019 Nov 28;24(2):1700–12. doi: 10.1111/jcmm.14863 (PMC6991678; doi:10.1111/jcmm.14863)

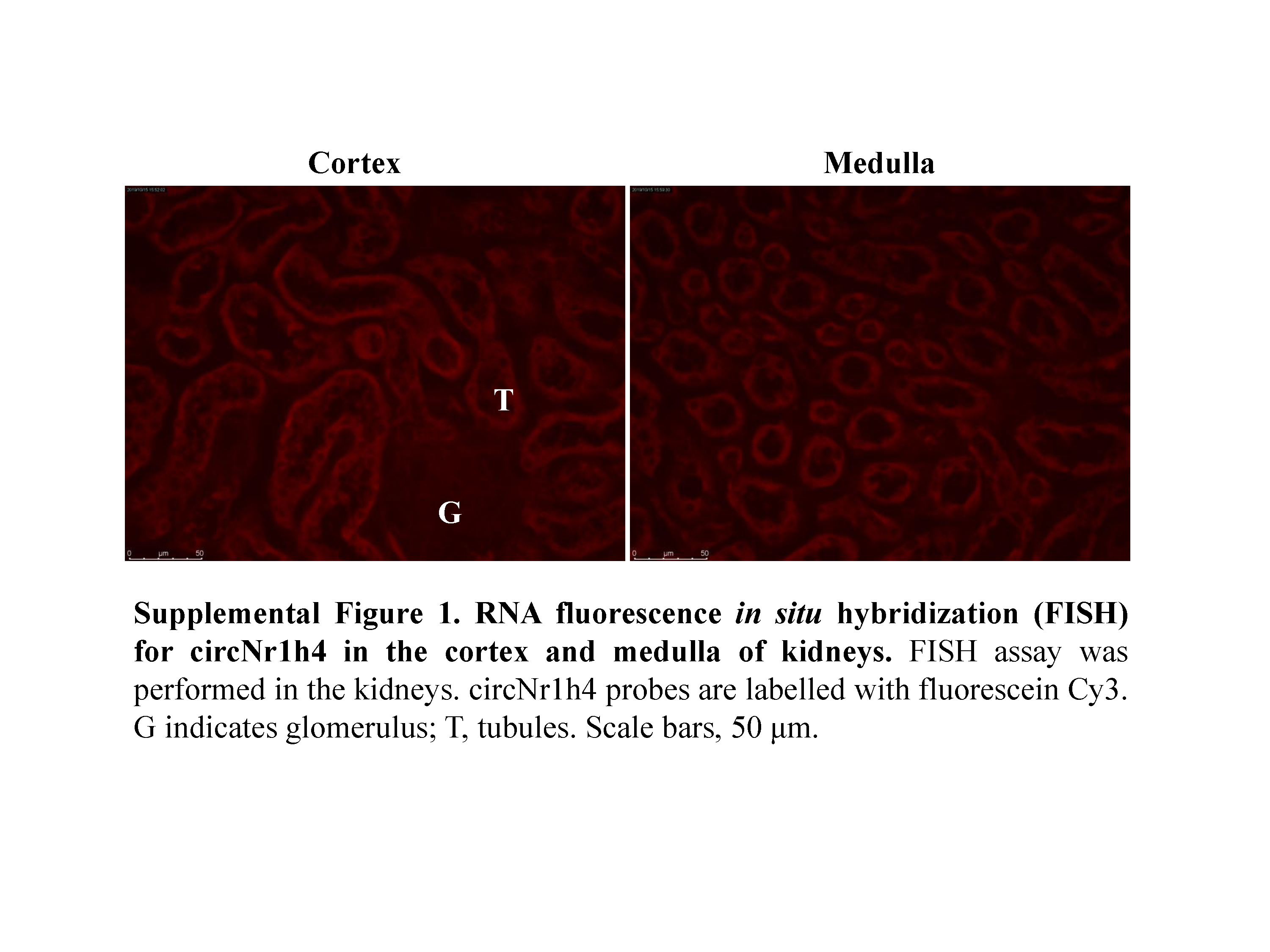

Supplement: Supplementary file 1 [file JCMM-24-1700-s001.tif]

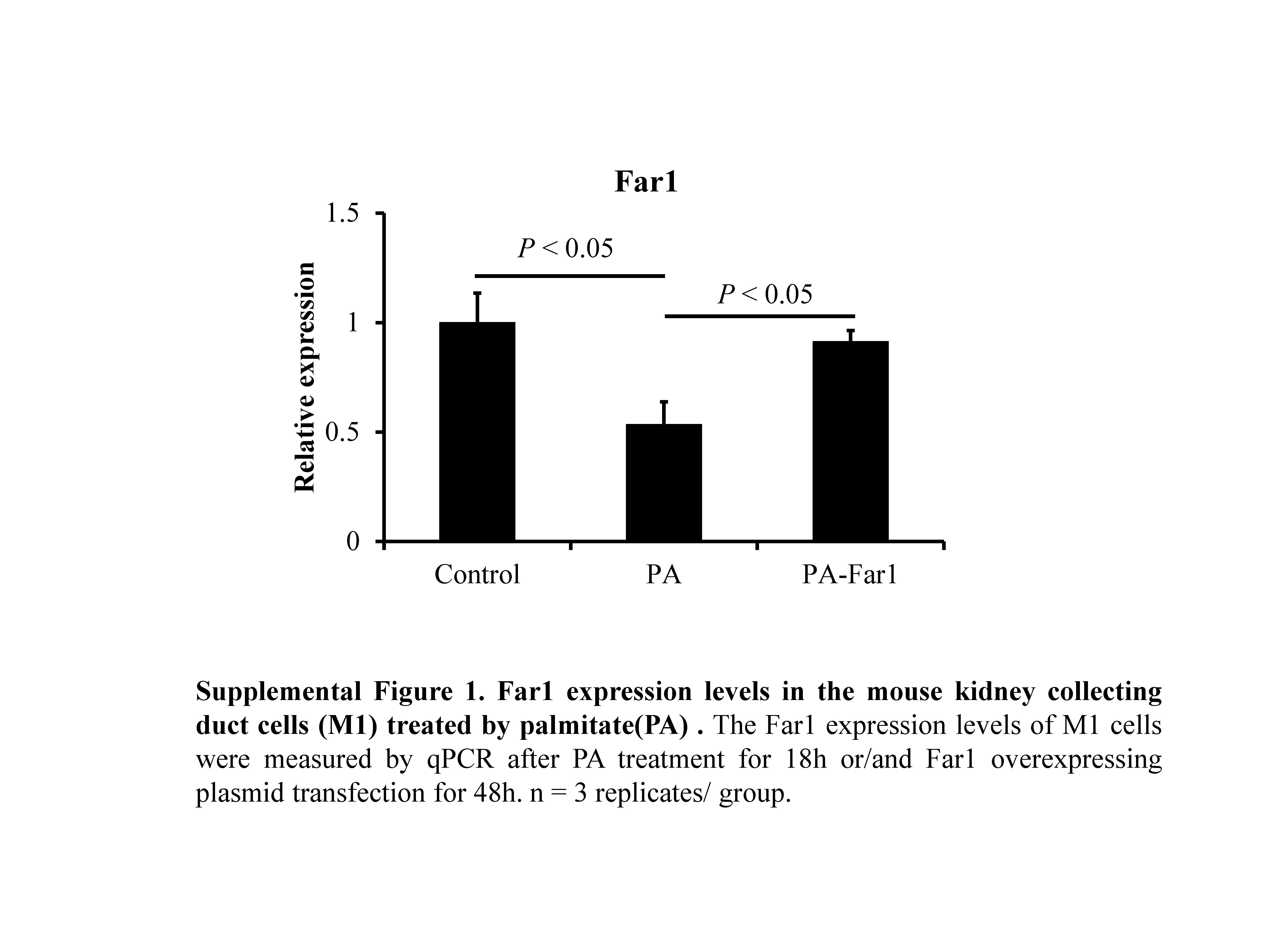

Supplement: Supplementary file 2 [file JCMM-24-1700-s002.tif]
