# Supplementary material for: CircNr1h4 regulates the pathological process of renal injury in salt‐sensitive hypertensive mice by targeting miR‐155‐5p
Source: J Cell Mol Med. 2019 Nov 28;24(2):1700–12. doi: 10.1111/jcmm.14863 (PMC6991678; doi:10.1111/jcmm.14863)
Supplement: Supplementary file 3 [file JCMM-24-1700-s003.docx]

Table 1 Summary of reads mapping to the *Mus musculus* reference genome

| Samples | Control1 | Control2 | Control3 | Doca1 | Docat2 | Doca3 |
| --- | --- | --- | --- | --- | --- | --- |
| Raw Reads | 104277476 | 97349202 | 90889254 | 93752956 | 100474714 | 100408696 |
| Valid reads | 98223312 | 90012742 | 84466932 | 88428226 | 94855962 | 94754016 |
| Mapped reads | 87512016 | 79837051 | 72806042 | 76909330 | 84943384 | 84443995 |
| Mapping ratio | 89.09% | 88.70% | 86.19% | 86.97% | 89.55% | 89.12% |
| Uniquely Mapped reads | 86300571 | 78141199 | 71536657 | 75689637 | 83722560 | 83154761 |
| Unique mapping ratio | 87.86% | 86.81% | 84.69% | 85.59% | 88.26% | 87.76% |
| Back-spliced junctions reads | 195780 | 179531 | 165977 | 490674 | 226108 | 332866 |
| Back-spliced junctions ratio | 0.20% | 0.20% | 0.20% | 0.55% | 0.24% | 0.35% |
