# Supplementary material for: CircNr1h4 regulates the pathological process of renal injury in salt‐sensitive hypertensive mice by targeting miR‐155‐5p
Source: J Cell Mol Med. 2019 Nov 28;24(2):1700–12. doi: 10.1111/jcmm.14863 (PMC6991678; doi:10.1111/jcmm.14863)
Supplement: Supplementary file 5 [file JCMM-24-1700-s005.docx]

Table 2 The top 10 most up-regulated circRNAs at the DOCA-salt mice compared to the control mice

| CircRNA ID | DOCA-salt  (FPKM) | Control  (FPKM) | log2  (fold change) | P-value | Host gene |
| --- | --- | --- | --- | --- | --- |
| circRNA267 | 4338.39 | 256.15 | 4.08 | 0.01 | Spp1 |
| circRNA958 | 14.38 | 1.06 | 3.77 | 0.00 | Aldh1a2 |
| circRNA962 | 12.46 | 1.72 | 2.86 | 0.03 | Col12a1 |
| circRNA779 | 1.81 | 0.29 | 2.65 | 0.01 | Kif1a |
| circRNA1836 | 0.79 | 0.13 | 2.60 | 0.01 | Kif14 |
| circRNA828 | 2.76 | 0.47 | 2.57 | 0.00 | Cenpf |
| circRNA4247 | 2.23 | 0.42 | 2.41 | 0.00 | Synpo |
| circRNA1045 | 25.51 | 5.15 | 2.31 | 0.00 | Dcdc2a |
| circRNA2251 | 2.88 | 0.65 | 2.15 | 0.00 | Mroh3 |
| circRNA3271 | 6.89 | 1.81 | 1.93 | 0.01 | Adgre1 |
