# Supplementary material for: CircNr1h4 regulates the pathological process of renal injury in salt‐sensitive hypertensive mice by targeting miR‐155‐5p
Source: J Cell Mol Med. 2019 Nov 28;24(2):1700–12. doi: 10.1111/jcmm.14863 (PMC6991678; doi:10.1111/jcmm.14863)
Supplement: Supplementary file 6 [file JCMM-24-1700-s006.docx]

Table 3 The top 10 most down-regulated circRNAs at the DOCA-salt mice compared to the control mice.

| CircRNA ID | DOCA-salt (FPKM) | Control (FPKM) | log2 (fold change) | P-value | Host gene |
| --- | --- | --- | --- | --- | --- |
| circRNA3498 | 9.95 | 141.50 | -3.83 | 0.01 | Slco1a1 |
| circRNA3161 | 0.34 | 1.94 | -2.53 | 0.01 | Kcnh5 |
| ciRNA57 | 2.16 | 10.46 | -2.27 | 0.01 | Amacr |
| circRNA2830 | 18.22 | 79.98 | -2.13 | 0.01 | Cyp2j13 |
| circRNA344 | 3.64 | 14.88 | -2.03 | 0.00 | Cyp2j7 |
| circRNA3990 | 0.23 | 0.92 | -2.02 | 0.03 | 4921507P07Rik |
| circRNA1806 | 4.52 | 17.93 | -1.99 | 0.03 | Cyp2j9 |
| circRNA2748 | 4897.41 | 18255.14 | -1.90 | 0.00 | Kap |
| circRNA3555 | 42.57 | 149.57 | -1.81 | 0.00 | Cyp2j13 |
| circRNA3538 | 5.50 | 19.17 | -1.80 | 0.05 | Glt1d1 |
