# Supplementary material for: CircNr1h4 regulates the pathological process of renal injury in salt‐sensitive hypertensive mice by targeting miR‐155‐5p
Source: J Cell Mol Med. 2019 Nov 28;24(2):1700–12. doi: 10.1111/jcmm.14863 (PMC6991678; doi:10.1111/jcmm.14863)
Supplement: Supplementary file 8 [file JCMM-24-1700-s008.docx]

Table 4 Differential expression patterns of circRNAs and mRNA from their parental host genes

| circRNA expression pattern | mRNA expression pattern | Genes in the pattern | Numbers of genes |
| --- | --- | --- | --- |
| DOCA > Control (62/11) | DOCA > Control | Smtnl2, Serpine2, Nuak2, Npnt, Kcnq1, Dcdc2a, Col12a1, Cenpf, Aldh1a2, Adgre1 | 10 |
|  | No significant differences | Wdhd1, Tmeff1, Synpo, Stat3, Spp1, Slc13a1, Slc12a4, Sla, Sh2d4b, Sgk1, Samd4, Rtn4, Rftn1, Rcn1, Rad51c, Pvt1, Ptpn13, Ppm1h, Pea15a, Pappa, Nckap5, Nav3, Mroh3, Mboat1, Lrrk1, Klf6, Kif26b, Kif1a, Igf2bp1, Gucy1a2, Gm28309, Ctsk, Csmd1, Cp, Cfh, Ccdc171, Anxa5, Adam12, 2610507B11Rik | 47 |
| DOCA < Control (62/8)) | DOCA < Control | Slco1a1, Slc22a22, Cyp2j7, Cyp2j13, Cd36, Ass1, Amacr | 7 |
|  | No significant differences | Tax1bp1, Tmem56, Tc2n, Sugct, Smarca2, Slc9a8, Slc27a2, Sepp1, Pik3c2g, Pank1, Nr1h4, Mlxipl, Mep1b, Malrd1, Lrrc69, Kcnh5, Kap, Gys2, Gm853, Glt1d1, Dnajc6, Dhtkd1, Cyp7b1, Cyp2j9, Crot, Cr2, Cpeb3, C330002G04Rik, Atp11a, Aspa, Agps, 9530026P05Rik, 4921507P07Rik | 35 |
