# Supplementary material for: CircNr1h4 regulates the pathological process of renal injury in salt‐sensitive hypertensive mice by targeting miR‐155‐5p
Source: J Cell Mol Med. 2019 Nov 28;24(2):1700–12. doi: 10.1111/jcmm.14863 (PMC6991678; doi:10.1111/jcmm.14863)
Supplement: Supplementary file 11 [file JCMM-24-1700-s011.docx]

**Supplemental Materials and Methods**

**Quantitative reverse-transcription polymerase chain reaction (qPCR)**

We performed qPCR to validate the results from the RNA sequencing approach. The Bulge-Loop^TM^ miRNA qPCR Primer Set (RiboBio, Guangzhou, China) was used to determine the expression levels of miRNAs by qRT-PCR according to the manufacturer’s instructions. U6 was used as an internal control. For circRNAs, we analyzed the predicted circRNAs using divergent primer by qPCR. For Far1, Cyp2j13, Ztp111, Glum, and Klhdc10 were performed as we described. ^27, 28^ The results were normalized to control values of glyceraldehyde-3-phosphate dehydrogenase (GAPDH). The primer sequences used in this study are listed in Supplemental Table 1. Negative controls were included with every qPCR assay. The 7500 fast real-time PCR system (Applied Biosystems, CA) was used for amplification and detection. RNase R treatment was performed as follows: total RNA was incubated for 15 min at 37 °C with 5 units RNase R per μg RNA (Epicentre Technologies, Madison, WI), and cDNA was then synthesized with random primer.

**Western blotting**

Kidney tissue was homogenized in RIPA buffer (0.5% NP-40, 0.1% sodium deoxycholate, 150 mmol L^−1^ NaCl, and 50 mmol L^−1^ Tris-Cl; pH 7.5). After centrifugation at 15 000 × g for 10 minutes at 4°C, the supernatant was collected and stored at −80°C. Lysates were resolved by SDS-PAGE, transferred to a PVDF membrane (Milipore, Billerica, MA), and probed with anti-Far1 (Abcam, Cambridge, UK) and anti-Gapdh antibodies (Santa Cruz Biotechnology, Santa Cruz, CA).

**Pull-down assay with a biotinylated DNA probe**

A biotinylated DNA probe complementary to circNr1h4 was synthesized and dissolved in 500 μl of binding/washing buffer (1 M NaCl, 5 mM Tris-HCl, 0.1% Triton X-100 and 0.5 mM EDTA; pH 7.5). The probes were incubated with streptavidin-coated magnetic beads (Invitrogen) at 25°C for 30 min to generate probe-coated magnetic beads. M1 cells were incubated in lysis buffer (20 mM Tris, pH7.5, 200 mM NaCl, 2.5 mM MgCl2, 0.05% Igepal, 1mM DTT, 200 U/mL RNase inhibitor, protease inhibitor) on ice and freeze-thawed for three times. The lysates were precleared by centrifugation, and 50 μl of the samples were aliquoted for input. The remaining lysates were incubated with M-270 streptavidin magnetic beads bound with a biotinylated DNA probe (RiboBio, Guangzhou, China). To prevent nonspecific binding of RNA, the beads were coated with yeast tRNA (Sigma). The beads were incubated at 4°C for 3h, washed twice with ice-cold lysis buffer, and then washed three times. The bound RNAs were purified using TRIzol for further analysis.
